# Responses of vegetation-soil enzyme activities to climatic factors under different treatments in desertified areas

**DOI:** 10.3389/fpls.2026.1827397

**Published:** 2026-05-19

**Authors:** Zhiting Wang, Tingxi Liu, Xin Tong, Yongzhi Bao, Mingyang Li, Limin Duan, Lina Hao, Tianyu Jia, Simin Zhang, Jiahao Sun

**Affiliations:** 1 State Key Laboratory of Water Engineering Ecology and Environment in Arid Area, Inner Mongolia Agricultural University, Hohhot, China; 2 College of Water Conservancy and Civil Engineering, Inner Mongolia Agricultural University, Hohhot, China; 3 Inner Mongolia Key Laboratory of Ecohydrology and High-Efficient Utilization of Water Resources, Hohhot, China; 4 Autonomous Region Collaborative Innovation Center for Integrated Management of Water Resources and Water Environment in the Inner Mongolia Reaches of the Yellow River, Hohhot, China; 5 Water Resources Research Institute of Shandong Province; Shandong Key Laboratory of Water Network Dispatching and Efficient Utilization, Jinan, China; 6 Institute of Water Sciences, Zhejiang University of Water Resources and Electric Power, Hangzhou, China

**Keywords:** climate conditions, dune, soil enzyme activity, treatment method, vegetation

## Abstract

**Introduction:**

Understanding the response mechanisms of vegetation characteristics and soil enzyme activities to climatic factors in desertified regions is crucial. However, the relationships between vegetation, soil enzyme activities, and climatic factors in dune ecosystems under different anthropogenic disturbances remain unclear.

**Methods:**

This study, based on *in situ* observations conducted from 2020 to 2023 in the Horqin Sand Land, systematically elucidates the response mechanisms of vegetation characteristics and soil enzyme activities to precipitation and temperature across different types of sand dunes under enclosure (control), mowing, burning, and grazing treatments.

**Results:**

The results indicate that the effects of mowing and burning on vegetation and soil enzyme activities are strongly water-dependent. In sand dunes located in the moisture-rich dune-meadow ecotone, these treatments exhibited compensatory effects, whereas in moisture-limited semi-mobile dunes, they led to inhibitory effects. Grazing, on the other hand, significantly reduced dune vegetation biomass and soil enzyme activities by 9-45%, but markedly increased plant species richness. Correlation analysis revealed that, following mowing and burning, Poaceae species in ecotone dunes were able to rapidly utilize precipitation, exhibiting high sensitivity to rainfall. In semi-mobile dunes where shrubs and herbaceous plants coexist, species with different life forms enhanced their relationships with precipitation through stratified water use. In contrast, grazing damaged vegetation via herbivory and trampling, overall weakening the response of vegetation to both temperature and precipitation. Structural equation modeling revealed that in ecotone dunes, precipitation significantly promoted sucrose enzyme activity only under burning and grazing treatments. In fixed dunes, however, due to the low palatability of Asteraceae species, livestock trampling facilitated litter decomposition and increased fecal inputs, thereby enhancing nutrient availability, and precipitation significantly promoted vegetation growth.

**Discussion:**

Overall, different disturbances modulated the responses of vegetation and soil enzyme activities to climatic factors by regulating soil hydrothermal conditions and resource use efficiency, highlighting the critical role of water availability in controlling ecological processes in desertified regions. This study provides a theoretical basis and technical support for the conservation and restoration of sandy ecosystems.

## 1 Introduction

Under the dual pressures of climate change and unsustainable human activities, the global ecological environment is facing multiple threats and severe challenges (Scanlon et al., 2023). Particularly in arid and semi-arid regions, desert grassland ecosystems with limited self-recovery capacity are experiencing increasingly severe degradation. Currently, desertified areas account for approximately 41% of the global land surface (Hu et al., 2022). Desertified areas affected by human activities are highly susceptible to sandstorms (Askhat et al., 2023), posing a serious threat to regional ecological security and sustainable development (Scanlon et al., 2023). Particularly in arid and semi-arid regions, desert grassland ecosystems with limited self-recovery capacity are experiencing increasingly severe degradation. Soil enzymes, as key regulators of soil biochemical processes and nutrient cycling, are highly sensitive to climate change and anthropogenic disturbances, and can serve as early indicators of changes in soil biological functioning (Wang et al., 2022b; Xu et al., 2021). Soil enzyme activities are particularly sensitive to the restoration of dune ecosystems (Qu and Hai, 2025), and mainly originate from root exudates, litter, and microbial activity (Xu et al., 2021). Soil enzyme activities catalyze the decomposition of organic matter and nutrient mineralization, releasing plant-available nutrients to meet energy demands (Ali et al., 2025; Zuccarini et al., 2023). The interaction mechanisms between vegetation and soil enzyme activities are complex, and this process is jointly regulated by precipitation, temperature, and anthropogenic disturbances (Castillo-Garcia et al., 2022; Fanin et al., 2022; Xu et al., 2021). Therefore, elucidating how climatic factors regulate vegetation and soil enzyme activities under different anthropogenic disturbances in desertified regions is of scientific and practical value for ecological restoration and sustainable development in semi-arid areas.

Numerous studies have investigated the effects of different anthropogenic disturbances on vegetation and soil properties, mainly focusing on enclosure (Xu et al., 2020), grazing (Dong et al., 2022), and mowing (Chen et al., 2021). Research on the effects of burning on vegetation and soil properties has mainly focused on natural burnings, whereas studies on controlled burns are relatively limited. The establishment of enclosures eliminates various anthropogenic disturbances, enhances soil fertility, and improves soil physicochemical properties, thereby promoting vegetation growth (Xu et al., 2020). The effects of enclosure on grassland vegetation and soil properties are related to the duration of enclosure. A study (Zhu et al., 2026) found that short-term enclosure helps improve soil fertility and vegetation growth, but prolonged enclosure reduces vegetation productivity and diversity. Grazing alters vegetation biomass and soil properties through herbivory, trampling, and fecal inputs, and its effects are primarily regulated by grazing intensity (Arroyo et al., 2024). A study (Li et al., 2025) found that increasing grazing intensity significantly reduced vegetation biomass and soil nutrient content. Mowing affects vegetation biomass by altering litter decomposition and light conditions, leading to the redistribution of soil nutrient resources (Chen et al., 2021). The effects of mowing on grassland vegetation and soil are related to mowing intensity, with severe mowing having a greater impact on vegetation and soil nutrients than light mowing (Mao et al., 2023). Overall, existing studies have largely focused on single types of disturbances and primarily on vegetation and soil nutrient characteristics, whereas research on soil enzyme activities under multiple anthropogenic disturbances remains relatively limited.

In arid ecosystems, in addition to anthropogenic disturbances, climatic factors are also key drivers influencing vegetation and soil enzyme activities (Cao et al., 2021). Studies have shown that climate warming can enhance photosynthetic rates and promote vegetation growth. However, it can also accelerate evapotranspiration and intensify soil drought, thereby exerting negative effects on vegetation (Yin et al., 2024). In arid environments, continuous warming may push vegetation drought tolerance beyond critical thresholds, thereby reducing plant diversity (Li et al., 2024b). Meanwhile, variations in precipitation can significantly alter soil wetting-drying cycles (Zaitchik et al., 2023). This process may disrupt soil aggregates and expose new soil surfaces; meanwhile, it can also uncover previously protected organic matter, thereby enhancing the availability of soil nutrients (Li et al., 2024a). Increased moisture can also supply more organic matter, thereby enhancing soil enzyme activities (Ma et al., 2020). In contrast, drought limits water availability, reduces soil moisture content, and suppresses microbial metabolism by disrupting hydrological processes, thereby constraining vegetation growth (Deng et al., 2021). However, some studies have found that drought does not significantly reduce soil microbial biomass, and this result is closely related to the drought tolerance of plants (Qu et al., 2023). Overall, the effects of precipitation and temperature on vegetation and soil enzyme activities are highly complex. However, studies focusing on desertified arid ecosystems remain limited. Existing research has largely concentrated on macroecological scales, and our understanding of how climatic factors drive changes in vegetation-soil enzyme activities at the microscale is still insufficient.

Given this, systematically evaluating the differences in vegetation-soil enzyme activities, and their responses to climatic factors under various anthropogenic disturbances in desertified regions has important implications for desertification control practices. In this study, ecotone, fixed, and semi-mobile dunes in northern China were selected as the study sites, with undisturbed grassland in each dune type serving as the control. During 2020-2023, experiments including mowing, burning, and grazing treatments were conducted, and vegetation characteristics and soil enzyme activities were systematically quantified under different treatments. With precipitation and temperature as the key driving factors, this study aims to explore under different treatments: (1) the variation patterns of vegetation-soil enzyme activities; (2) the response mechanisms of vegetation-soil enzyme activities to climatic factors; and (3) the driving mechanisms of vegetation-soil enzyme activities.

## 2 Materials and methods

### 2.1 Overview of the study area

**Figure 1:**
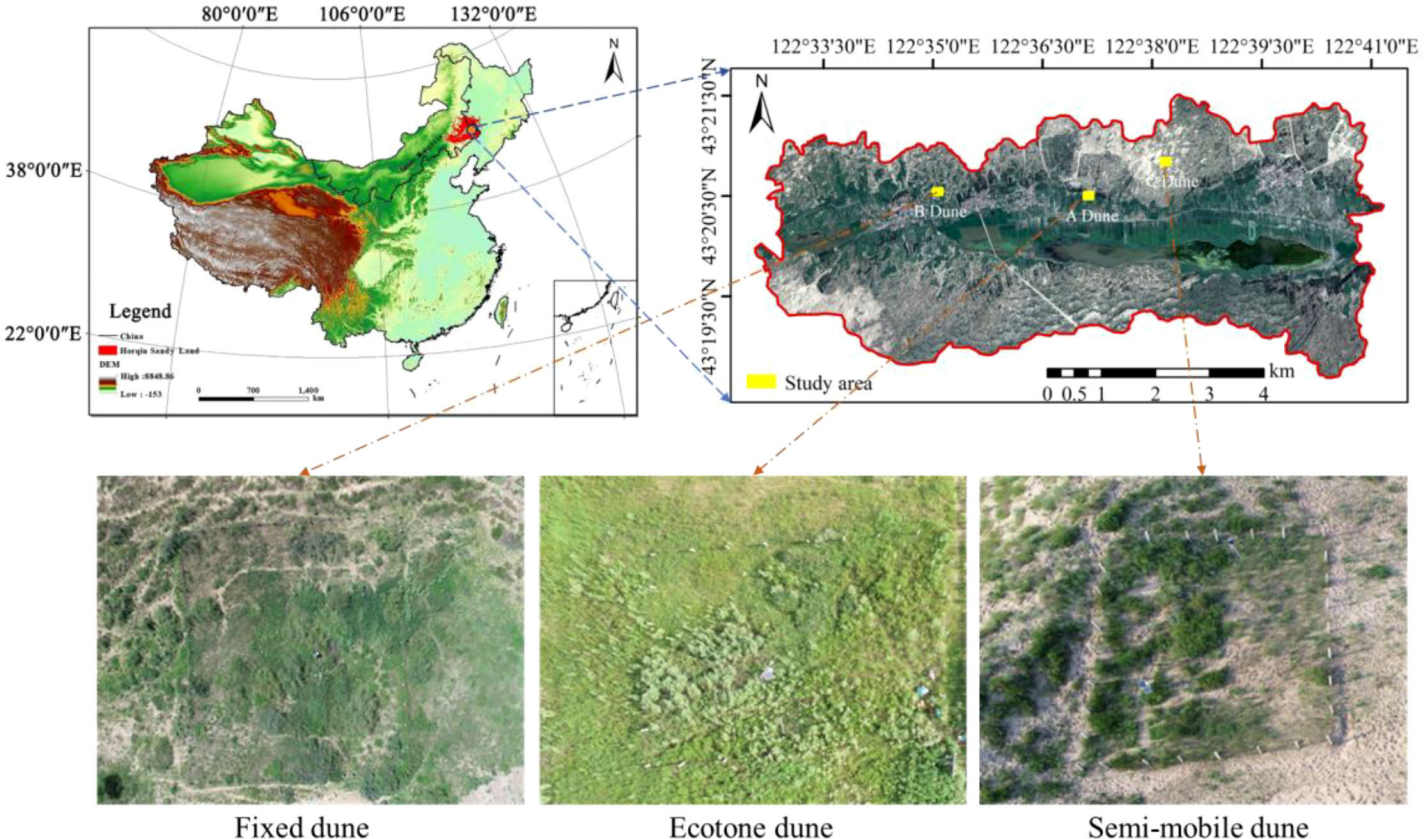
Schematic diagram of the layout of experimental points in the study area. A Dune, Ecotone dune; B dune, Fixed dune; C dune, Semi-mobile dune.

**Figure 2:**
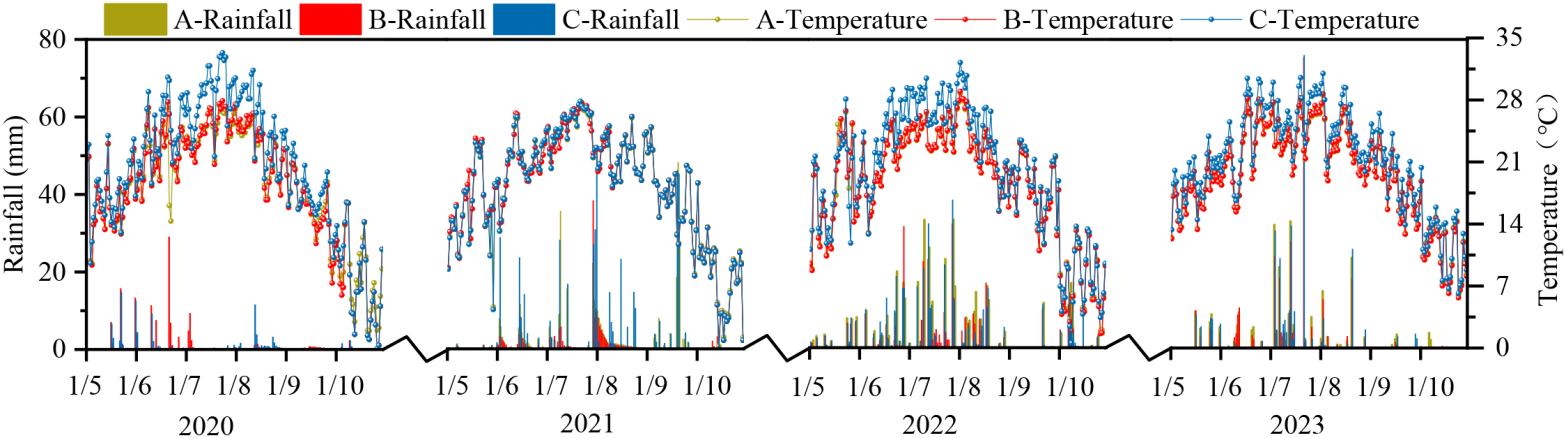
Changes in precipitation and temperature at different dunes in the study area from 2020 to 2023. A, Ecotone dune; B, Fixed dune; C, Semi-mobile dune.

The study area is located on the southeastern edge of the Horqin Sandy Land (122°33′00″-122°41′00″E, 43°18′48″-43°21′24″N). The region experiences a temperate continental monsoon climate, with the vegetation growing season spanning from May to October. The mean annual precipitation is 389 mm, with approximately 80% occurring between May and September. The mean annual temperature is 6.6 °C, and the mean annual evaporation reaches 1412 mm. A variety of landform types are distributed from north to south toward the central area, including dunes (mobile dunes, semi-mobile dunes, fixed dunes and ecotone dunes), meadow farmland, meadow grassland, and lakes, forming a distinctive dune-meadow mosaic. Soil moisture and vegetation coverage decreased sequentially from ecotone dunes to fixed dunes and semi-mobile dunes. The vegetation in ecotone dune primarily consists of Poaceae species such as *Phragmites australis*, *Stipa capillata*, and *Leymus chinensis*; the Fixed dune are dominated by Asteraceae species including *Artemisia frigida*, *Artemisia scoparia*, and *Artemisia sieversiana*; and Semi-mobile dune are characterized by semi-shrubs and herbs such as *Artemisia halodendron*, *Koenigia divaricata*, and *Grubovia dasyphylla*. The specific experimental location is shown in **Figure 1**, and the rainfall and temperature data from 2020 to 2023 for different dune types are presented in **Figure 2**.

### 2.2 Plot survey

**Figure 3:**
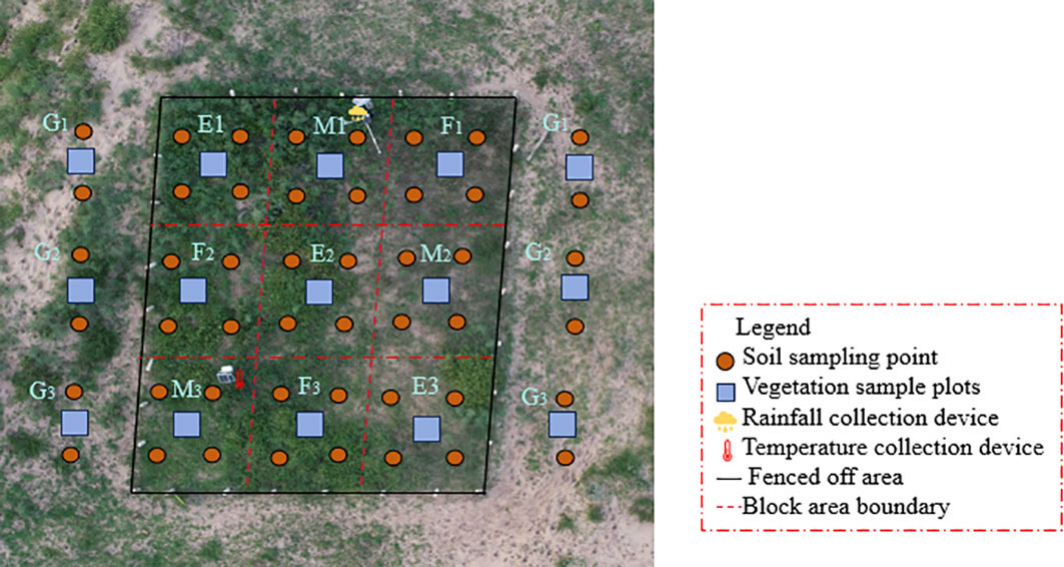
Schematic diagram of different treatment methods for dune grassland. E, Enclosure; M, Mowing; F, Burning; G, Grazing.

Beginning in 2020, three dune types (**Figure 1**) were selected as study sites. In 2010, a 1600 m² (40 m × 40 m) grassland plot was fenced to exclude human activity, allowing vegetation to grow naturally. For each dune, a control plot (E, CK) was established, with each plot covering one third of the area of the enclosure plot. No anthropogenic disturbance was applied to the control plots, and vegetation was allowed to grow under natural conditions. In August 2019, this study employed a completely randomized block design, with three experimental plots evenly established within each enclosure: control treatment (E), mowing treatment (M), and burning treatment (F). Each treatment plot included three replicates, with each plot occupying approximately one-third of the total enclosure area (**Figure 3**). In the control plots, no anthropogenic disturbances were applied, allowing vegetation to grow naturally. From 2020 to 2023, mowing was conducted annually in August after seed maturation. One-third of the grassland area within each fenced plot was mowed at ground level using sickles, leaving a stubble height of 7 cm. All plant residues from the mowed plots were removed using rakes and transported out of the plots. This treatment was defined as the mowing treatment (M). From 2020 to 2023, prescribed burning was conducted annually in April, one third of the grassland area within the fenced plots under clear and windless conditions (wind speed < 5 m/s). The burning was ignited at one corner of each plot and carried out as a controlled burning along a predetermined route. Due to the uniform distribution of standing dead vegetation and litter within the plots, the low-intensity burning rapidly spread across the entire area. Areas outside the enclosures served as grazing zones. Before grazing commenced, structured interviews were conducted with local herding households to obtain information on grazing management. Grazing plots were established in areas with stable grazing practices and a grazing history of over 20 years, with each plot measuring 100 × 100 m (1 ha). The grazing flocks consisted predominantly of adult sheep (90%), were grazed only during the daytime, and were provided with adequate water and salt supplements. Grazing occurred continuously from 08:00 to 18:00 daily, with full-time, uninterrupted grazing and no resting period. Each day, the sheep were driven by herders to the experimental plots for free grazing and returned to the pens in the evening, with ad libitum access to water and mineral salt supplementation. Grazing intensity was calculated based on the annual average stocking rate, which was 10 SU·ha^-^¹·a^-^¹ (SU, sheep unit; one standard unit corresponds to a 50 kg sheep), representing sustained high-intensity grazing over multiple years. During grazing, GPS collars were used to track the movement paths of the sheep, and fecal deposition was regularly monitored using a fixed-point counting method. All the treatment areas shared identical soil types and geographic conditions and included a 1m buffer zone. In early April 2020, each treatment area was subdivided into three blocks. On April 2020-2023, one to two 2 m × 2 m vegetation plots and four soil sampling points were established within each block, resulting in 12 soil sampling points and 3–6 vegetation plots per treatment (**Figure 3**).

### 2.3 Vegetation and soil sampling and testing

#### 2.3.1 Vegetation ecological indicators

The vegetation survey was conducted annually during the growing season (May-August) from 2020 to 2023. Aboveground biomass is defined as the dry weight of living and dead plant materials per unit area, representing the energy and matter accumulated through photosynthesis by green plants. Aboveground vegetation biomass was determined using the harvest-and-weigh method. Within each quadrat, aboveground plant materials were clipped at ground level, sorted by vegetation type (species), and labeled. The samples were then transported to the laboratory and dried in a thermostatic electric drying oven (DHG-9030A, USA): first at 105 °C for 30 min to deactivate enzymes, and subsequently at 65 °C for 48 h until constant mass was achieved. Finally, samples were weighed to obtain dry mass, and aboveground biomass was calculated on an area basis by converting the dry mass to unit ground area (g m^-^²) according to the quadrat area. Plant species richness (R) represents the number of plant species within a quadrat. The calculation formulas for aboveground biomass (AGB, Equation 1) (Bazrafkan et al., 2023) and plant species richness (R, Equation 2) (Wang et al., 2024) are as follows:

(1) $$\begin{array}{c} AGB=\frac{W}{A}({\text{gm}}^{-2}) \end{array}$$

(2) $$\begin{array}{c} R=S \end{array}$$

where *W* represents the dry weight of plant materials within a quadrat (g), *A* represents the quadrat area (m²), and *S* represents the number of vegetation species within the quadrat.

#### 2.3.2 Soil enzyme activity

At the end of each vegetation survey, soil samples were collected to assess soil enzyme activity. Prior to sampling, the surface litter was removed, and the soil cores were extracted at three depths (0-5, 5-10, and 10–20 cm) using a soil auger with a 7 cm diameter. Three replicate samples were collected at each sampling point. Samples from the same depth, block, and treatment were thoroughly homogenized, placed in pre-labeled self-sealing bags, transported to a shaded indoor environment, air-dried, and passed through a 30-mesh sieve to remove large stones and root debris. Soil enzyme activities were determined using commercial soil enzyme activity assay kits (Solarbio Science & Technology Co., Ltd., China) following the microplate method. All procedures were performed strictly according to the manufacturer’s instructions. Soil enzyme activities were measured using UV-3600 ultraviolet spectrophotometer (Shimadzu Co., Ltd., Japan).

### 2.4 Meteorological data

Meteorological data, including temperature and rainfall, were collected for different sand dune types from 2020 to 2023. A meteorological monitoring device was installed at each site. Rainfall was measured using a tipping bucket rain gauge (TE525MM, Campbell Scientific, Inc., USA), while air temperature was recorded using a temperature sensor (HMP45C, Campbell Scientific, Inc., USA) mounted at a height of 2 m. The data were logged at the 10-minute intervals. The data were stored using an automatic data logger. Air temperature data were calculated as daily mean values, while precipitation was calculated as daily totals. The daily dynamics of air temperature and precipitation from May to August during 2020–2023 are shown in **Figure 2**.

### 2.5 Data analysis

Differences among treatments were evaluated using one-way ANOVA followed by Duncan’s multiple range test. Significance testing was conducted using the least significant difference (LSD) method, with the significance level set at P < 0.05 (Wang et al., 2025). Correlation analysis was conducted using the Pearson correlation coefficient method (Wang et al., 2025). At the 95% confidence level, bivariate Pearson correlation coefficients were used to test the relationships among variables under different treatments (Zhong et al., 2022). To further explore the mechanisms by which driving variables influence target variables, a structural equation modeling (SEM) framework was employed. SEM analyses were performed using AMOS 27.0 (IBM Corp., Armonk, NY, USA) to evaluate both the direct and indirect effects of the driving variables (Gu et al., 2022). Model goodness-of-fit was assessed using the comparative fit index (CFI), goodness-of-fit index (GFI), and chi-square value (χ²) (Gu et al., 2022). Generally, higher values of CFI and GFI (closer to 1) and lower χ² values (closer to 0) indicate a better model fit (Gu et al., 2022). All figures were generated using Origin 2021 (Origin Lab Corp., Northampton, MA, USA).

## 3 Results and analysis

### 3.1 Responses of vegetation ecological characteristics to different grassland treatments and climatic factors

**Figure 4:**
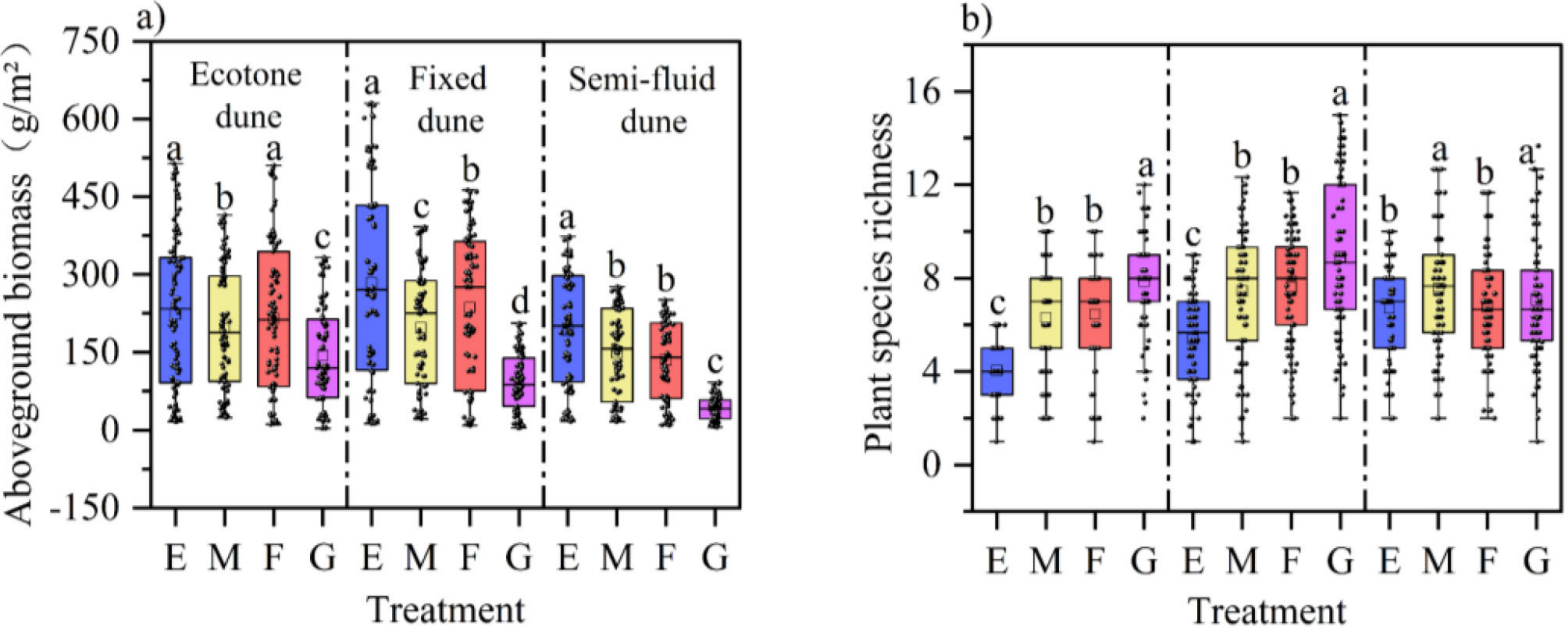
Variation in vegetation characteristics under different treatments. **(A)** Dune, Ecotone dune; **(B)** Dune, Fixed dune; C Dune, Semi-mobile dune; E, Enclosure; M, Mowing; F, Burning; G, Grazing.

The vegetation ecological responses under different treatments are shown in **Figure 4**. Compared with the enclosure treatment, mowing and grazing significantly reduced aboveground biomass across all dune types (12-36%), while markedly increasing plant species richness indices (36-79%; P < 0.05). The effects of burning on vegetation characteristics varied significantly among dune types. For aboveground biomass, burning had no significant effect on ecotone dunes (P > 0.05), but significantly reduced biomass on fixed and semi-mobile dunes (P < 0.05). Regarding plant species richness indices, burning significantly increased plant species richness on ecotone and fixed dunes (P < 0.05), whereas its effect on semi-mobile dunes was not significant (P > 0.05).

**Figure 5:**
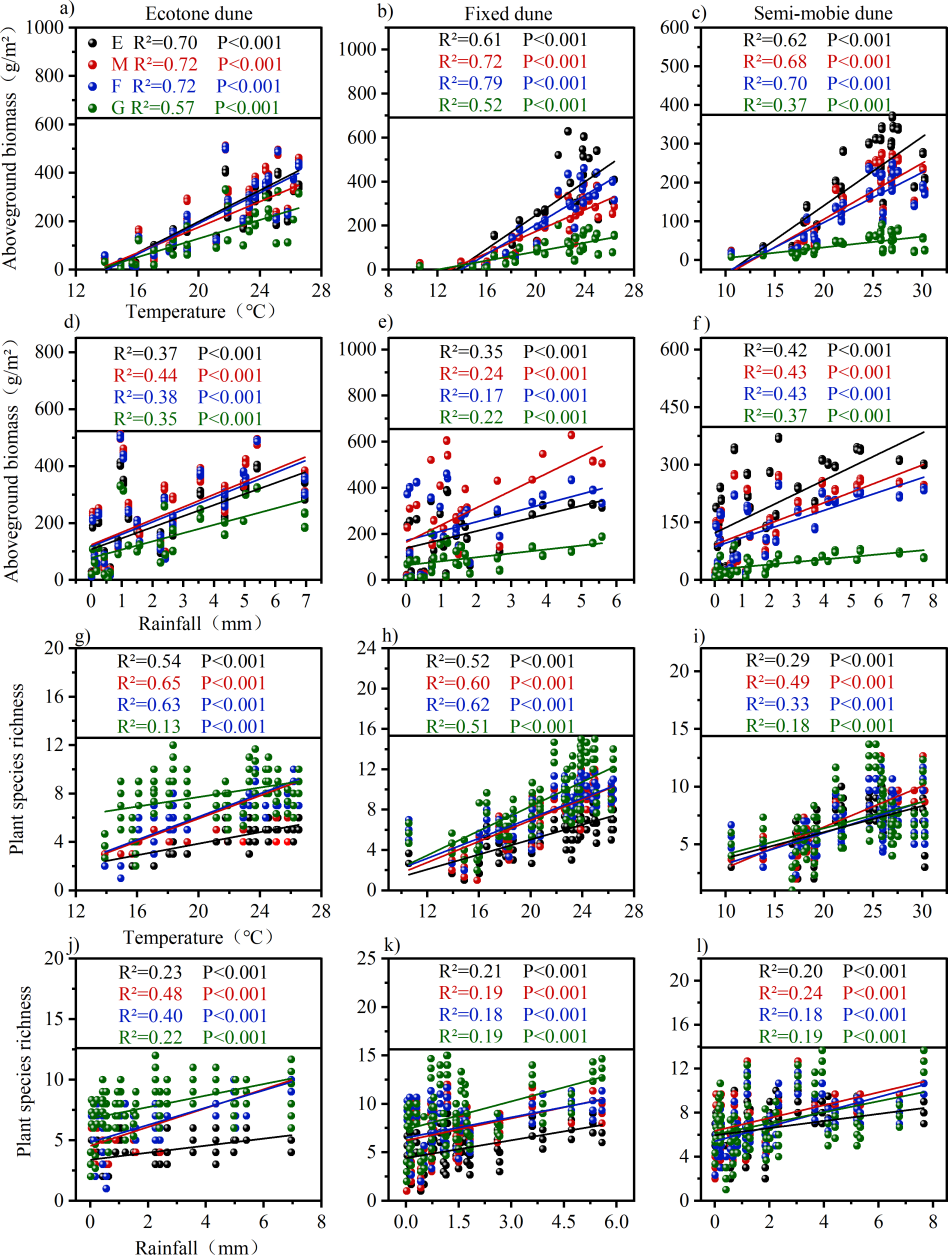
Effects of climatic factors on vegetation characteristics under different treatments. **(a-c)** represent the relationship between aboveground biomass and temperature in Ecotone dunes, fixed dunes, and semi-mobile dunes. **(d-f)** represent the relationship between aboveground biomass and Rainfall in Ecotone dunes, fixed dunes, and semi-mobile dunes. **(g-i)** represent the relationship plant species richness and temperature in Ecotone dunes, fixed dunes, and semi-mobile dunes. **(j-l)** represent the relationship plant species richness and rainfall in Ecotone dunes, fixed dunes, and semi-mobile dunes. E, Enclosure; M, Mowing; F, Burning; G, Grazing; Colored lines represent the linear fit for each sample group. R^2^ values, ranging from 0 to 1, indicate the strength of the correlation-the closer to 1, the stronger the correlation. P values indicate statistical significance: P < 0.001 indicates highly significant correlation, while P < 0.05 indicates significant correlation.

Anthropogenic disturbances altered the responses of vegetation to climatic factors. Mowing and burning enhanced the responses of vegetation to temperature across all dune types (**Figures 5a–c, g–i**), and strengthened their responses to precipitation in ecotone and semi-mobile dunes, whereas the opposite trend was observed in fixed dunes (**Figures 5d–f, j–l**). In contrast, grazing overall weakened the correlations between vegetation and both temperature and precipitation (**Figures 5a–l**), indicating that sustained grazing reduces vegetation responsiveness to climate change.

### 3.2 Response of soil enzyme activity to different treatments

**Figure 6:**
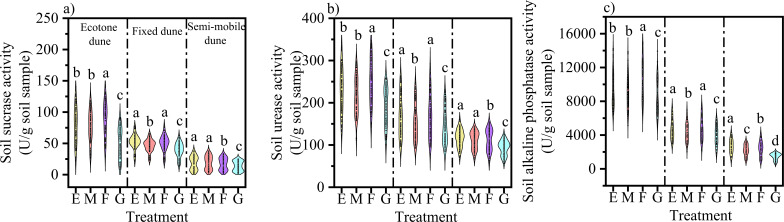
Effects of different treatments on soil enzyme activity. **(a-c)** represent the variations in soil sucrase, urease, and alkaline phosphatase. E, Enclosure; M, Mowing; F, Burning; G, Grazing; The top and bottom bars, box limits, and lines within the box represent the highest and lowest values, the 25-75th percentile range, and the mean, respectively. Different lowercase letters above the violins (a, b, c, d) indicate statistically significant differences among treatments (E, M, F, G) at P < 0.05.

The effects of different treatments on soil enzyme activities varied significantly among dune types (**Figure 6**). Overall, grazing consistently exhibited a significant inhibitory effect across all dune types (9-45%; P < 0.05). The impact of mowing varied depending on enzyme and dune type, primarily resulting in a significant reduction in enzyme activities on fixed dunes (7-12%; P < 0.05). In ecotone and semi-mobile dunes, soil sucrase and urease activities were not significantly affected by mowing (P > 0.05), whereas alkaline phosphatase activity on semi-mobile dunes was significantly inhibited (P < 0.05). In contrast, burning exhibited an opposite trend across dune types: it significantly promoted soil enzyme activities in ecotone dunes (P < 0.05), significantly inhibited them in semi-mobile dunes (20-45%; P < 0.05), and had no significant effect on fixed dunes (P > 0.05).

**Figure 7:**
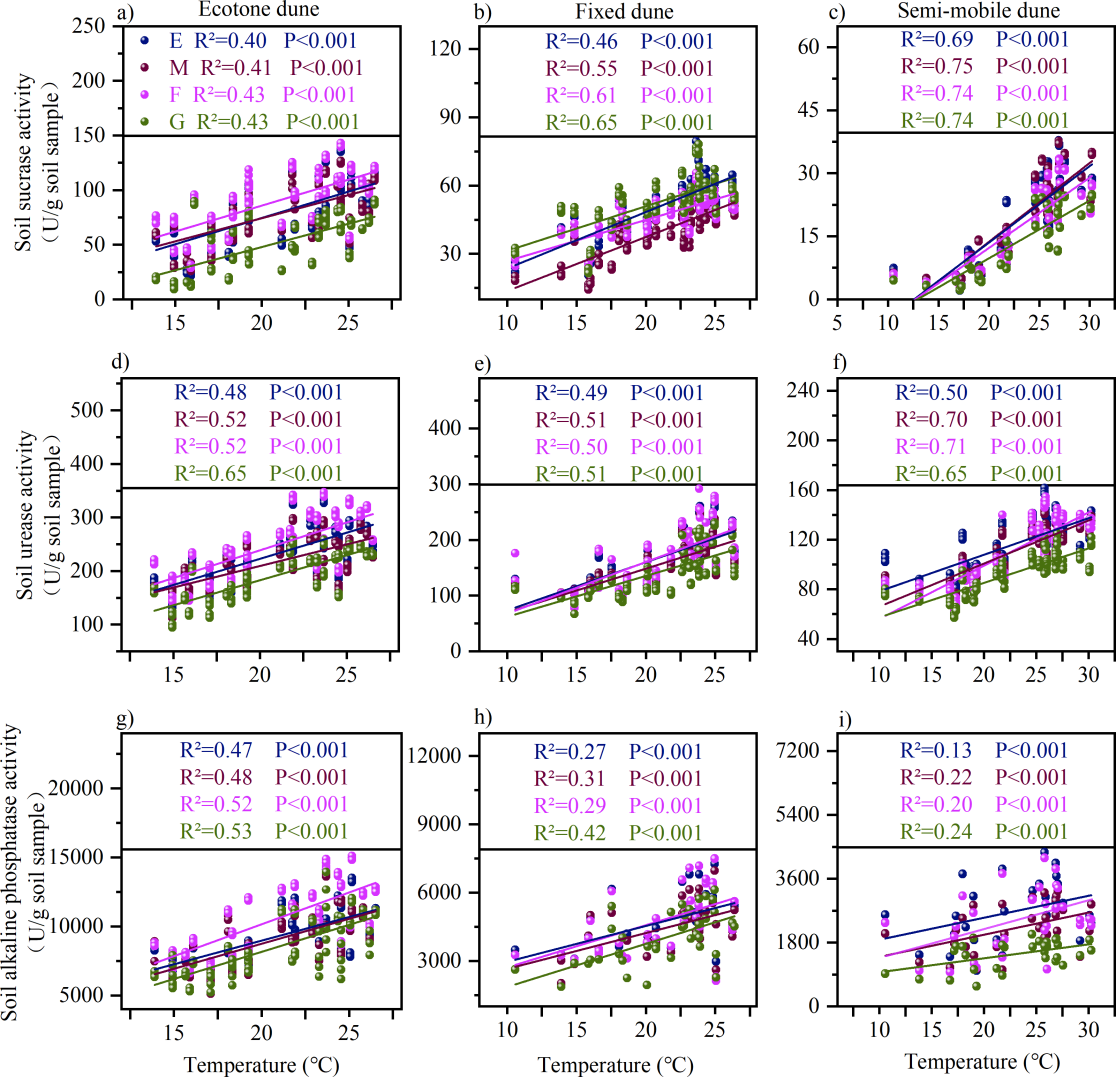
Relationship between air temperature and soil enzyme activity under different treatments. **(a-c)** illustrate the relationship between soil sucrase activity and temperature in Ecotone, fixed, and semi-mobile dunes; **(d-f)** depict the relationship between soil urease activity and temperature in Ecotone, fixed, and semi-mobile dunes; and **(g-i)** represent the relationship between soil alkaline phosphatase activity and temperature in Ecotone, fixed, and semi-mobile dunes. E, Enclosure; M, Mowing; F, Burning; G, Grazing; Colored lines represent the linear fit for each sample group. R^2^ values, ranging from 0 to 1, indicate the strength of the correlation-the closer to 1, the stronger the correlation. P values indicate statistical significance: P < 0.001 indicates highly significant correlation, while P < 0.05 indicates significant correlation.

**Figure 8:**
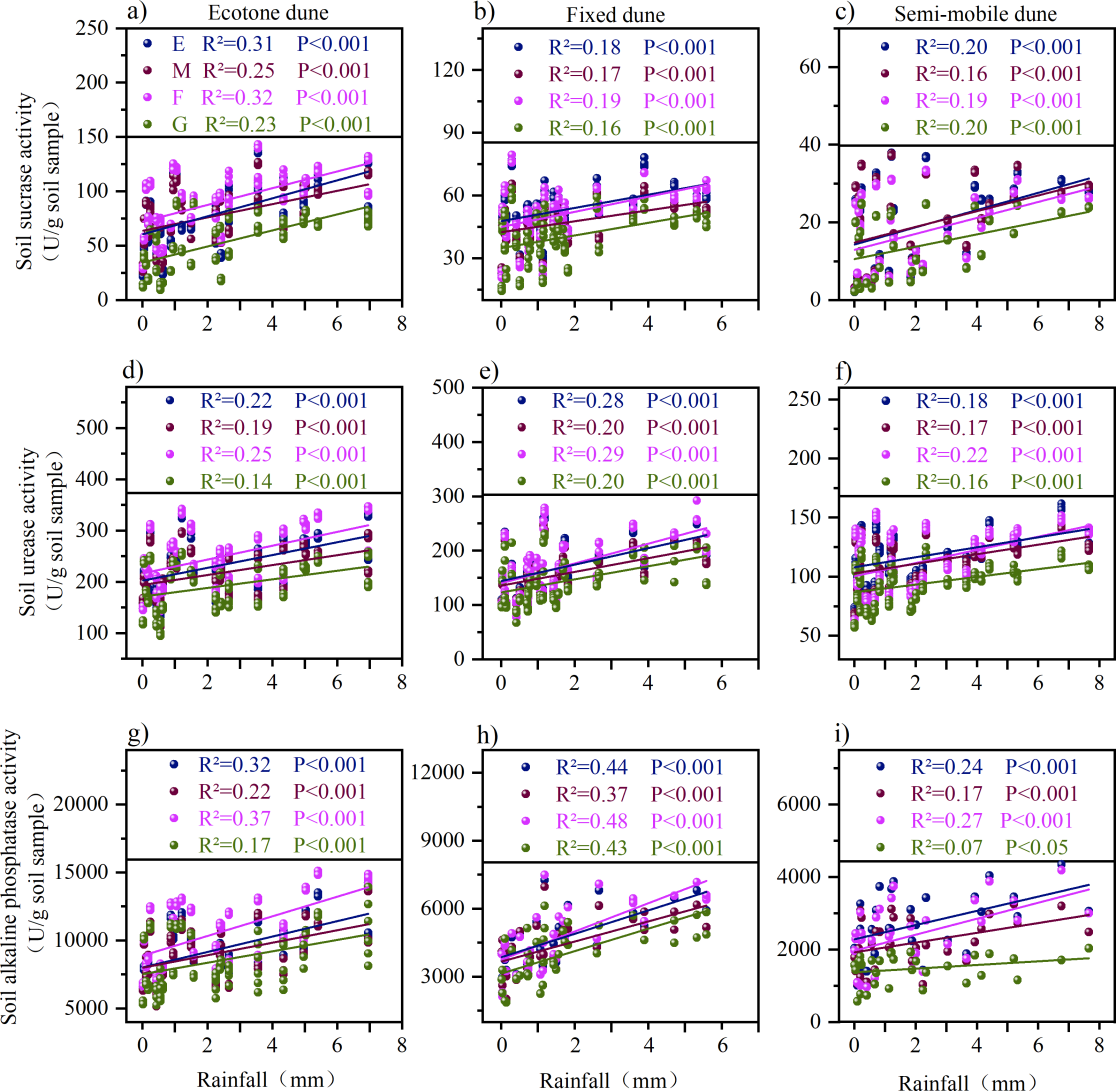
Relationship between rainfall and soil enzyme activity under different treatments. **(a-c)** illustrate the relationship between soil sucrase activity and rainfall in Ecotone, fixed, and semi-mobile dunes; **(d-f)** depict the relationship between soil urease activity and rainfall in Ecotone, fixed, and semi-mobile dunes; and **(g-i)** represent the relationship between soil alkaline phosphatase activity and rainfall in Ecotone, fixed, and semi-mobile dunes. E, Enclosure; M, Mowing; F, Burning; G, Grazing; Colored lines represent the linear fit for each sample group. R^2^ values, ranging from 0 to 1, indicate the strength of the correlation-the closer to 1, the stronger the correlation. P values indicate statistical significance: P < 0.001 indicates highly significant correlation, while P < 0.05 indicates significant correlation.

Soil enzyme activities in dunes were highly significantly correlated with both temperature and precipitation (**Figures 7**, **8**), and different disturbance treatments markedly altered their responses to these climatic factors. Specifically, regarding temperature, mowing, burning, and grazing all enhanced the responsiveness of soil enzyme activities to temperature fluctuations (**Figure 7**). In terms of precipitation, mowing and grazing weakened the correlations compared with the enclosure treatment, whereas burning exhibited a significant strengthening effect (**Figure 8**).

### 3.3 Relationship between vegetation ecological characteristics and soil enzyme activity

**Figure 9:**
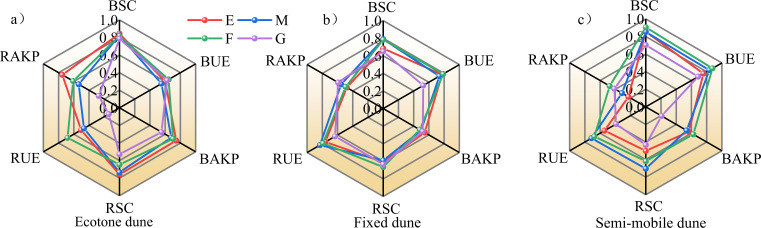
Correlation coefficients between vegetation factors and soil enzyme activities, and their responses to treatments. **(a-c)** represent the relationships between vegetation and soil enzyme activity in Ecotone, fixed, and semi-mobile dunes. E, Enclosure; M, Mowing; F, Burning; G, Grazing; BSC, BUE, and BAKP denote the correlation between aboveground biomass and soil sucrase, urease, and alkaline phosphatase activity, respectively; RSC, RUE, and RAKP denote the correlation of plant species richness with soil sucrase, urease, and alkaline phosphatase activity, respectively. R is between 0 and 1, the closer to 1, the better the correlation.

Compared with the enclosure treatment, grazing overall weakened the correlations between vegetation and soil enzyme activities. The effects of burning varied among dune types. On fixed (**Figure 9b**) and semi-mobile dunes (**Figure 9c**), burning enhanced the correlations between vegetation and soil enzyme activities, whereas on ecotone dunes (**Figure 9a**), it weakened the correlation between vegetation and alkaline phosphatase activity. The effects of mowing also exhibited dune-type dependency. Compared with the enclosure treatment, the correlation between vegetation and soil enzyme activities was generally weakened in ecotone dunes, whereas it was enhanced in semi-mobile dunes. Overall, the impacts of different disturbance treatments on vegetation-soil enzyme relationships were significantly dune-dependent, with grazing primarily exerting a weakening effect, while the direction of the effects of burning and mowing varied according to dune type.

### 3.4 Path analysis of soil enzyme activities and vegetation under climatic drivers across different treatments

**Figure 10:**
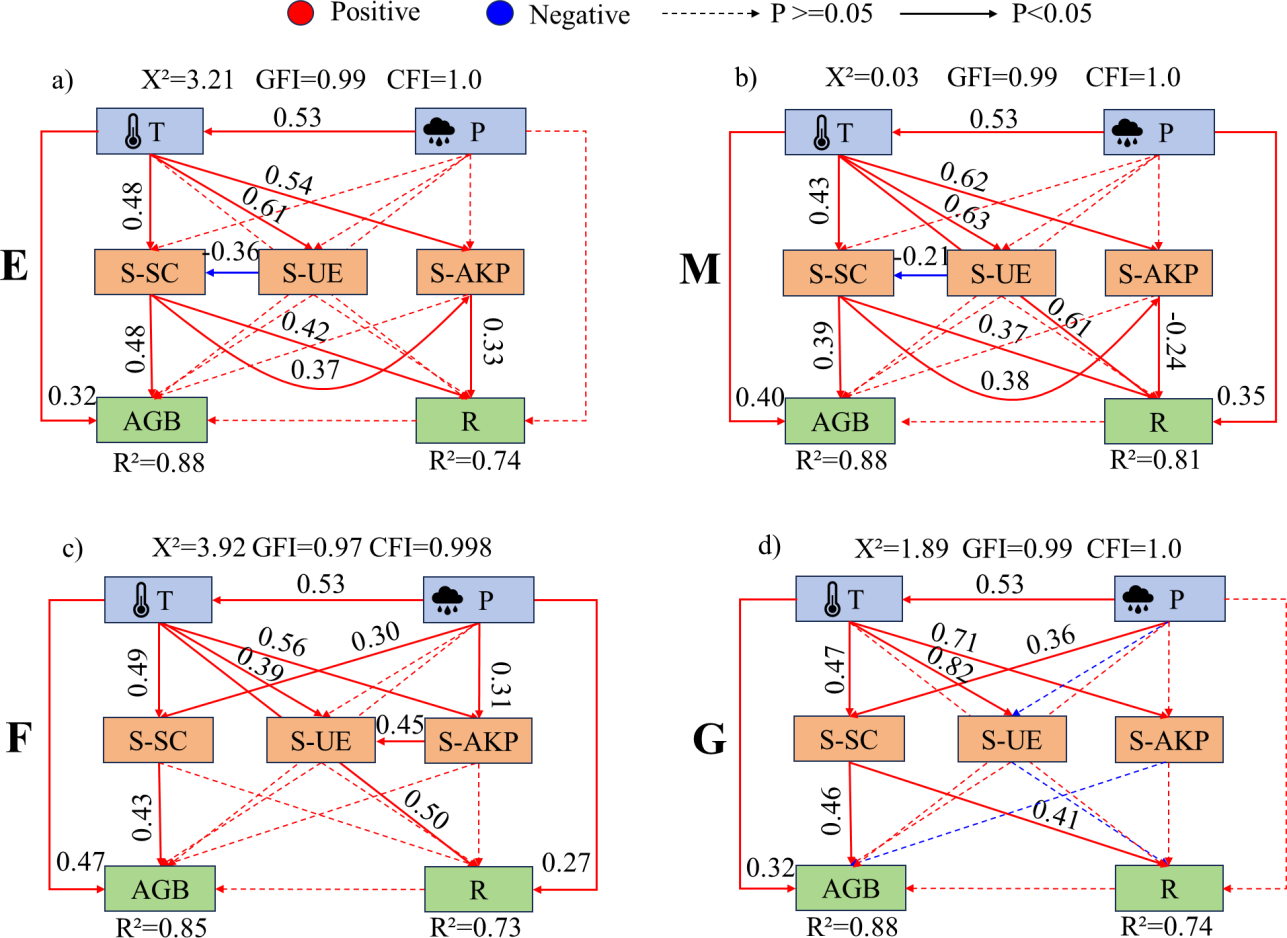
Structural equation model of the effects of climatic factors on soil enzyme activities and vegetation under different treatments in ecotone dunes. **(a-d)** represent the structural equation models illustrating the effects of climatic factors on soil enzyme activity and vegetation under enclosure, mowing, burning, and grazing treatments, respectively. E, Enclosure; M, Mowing; F, Burning; G, Grazing; T, Temperature; P, Rainfall; AGB, Aboveground biomass; R, plant species richness; S-SC, Soil sucrase activity; S-UE, Soil urease activity; S-AKP, Soil alkaline phosphatase activity. Red and blue arrows denote positive and negative relationships, respectively, while solid or dashed lines represent significant (p < 0.05) or non-significant relationships. Path coefficients are indicated near the arrows, showing the standardized path coefficients. R2 indicates the proportion of variance explained by each dependent variable. The goodness of fit of the model was evaluated using GFI, CFI, and X^2^ values. A better model fit is indicated when GFI and CFI approach 1, and the X^2^ value approaches 0.

**Figure 11:**
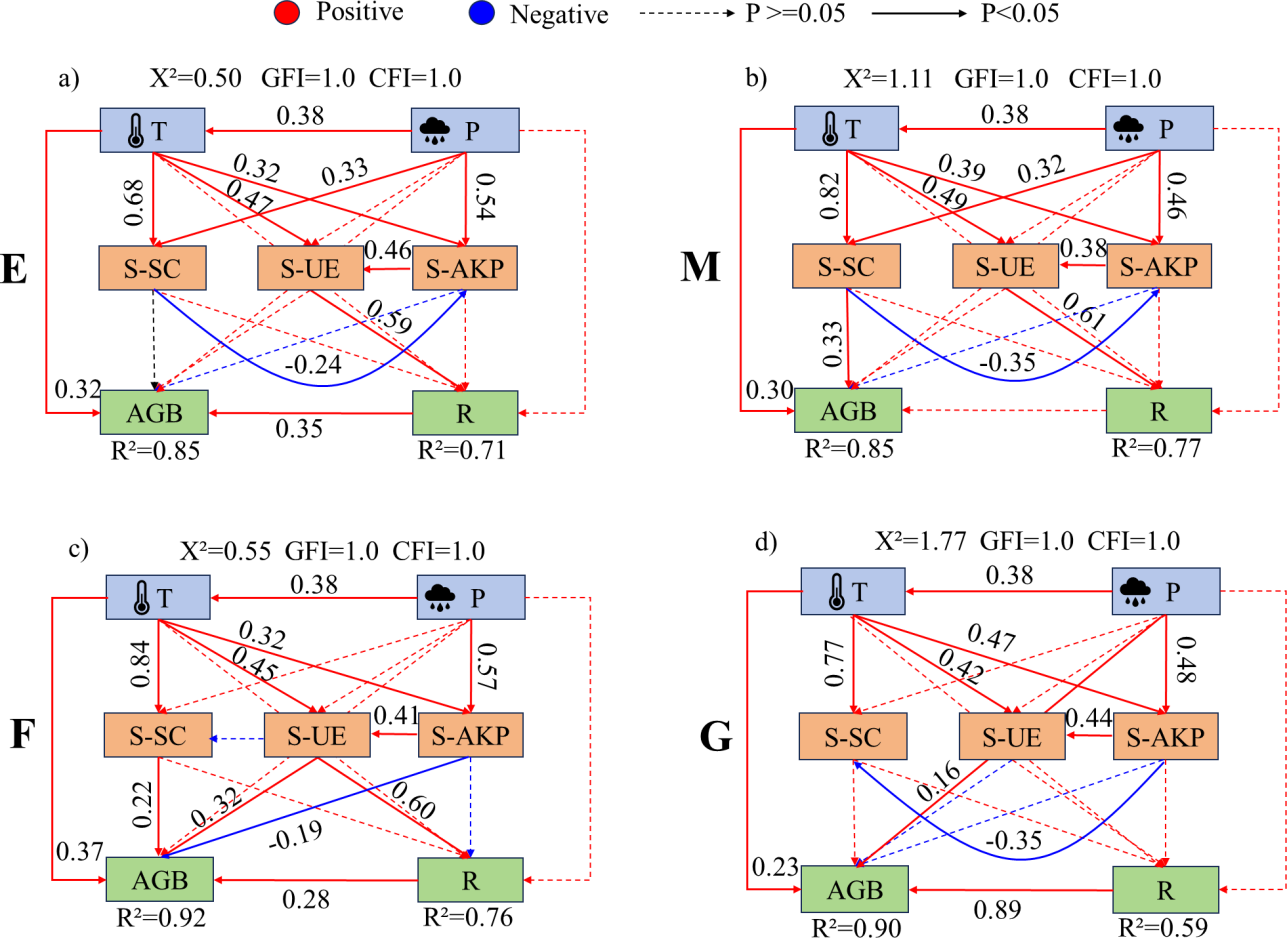
Structural equation model of the effects of climatic factors on soil enzyme activities and vegetation characteristics under different treatments in fixed dunes. **(a-d)** represent the structural equation models illustrating the effects of climatic factors on soil enzyme activity and vegetation under enclosure, mowing, burning, and grazing treatments, respectively. E, Enclosure; M, Mowing; F, Burning; G, Grazing; T, Temperature; P, Rainfall; AGB, Aboveground biomass; R, plant species richness; S-SC, Soil sucrase activity; S-UE, Soil urease activity; S-AKP, Soil alkaline phosphatase activity. Red and blue arrows denote positive and negative relationships, respectively, while solid or dashed lines represent significant (p < 0.05) or non-significant relationships. Path coefficients are indicated near the arrows, showing the standardized path coefficients. R2 indicates the proportion of variance explained by each dependent variable. The goodness of fit of the model was evaluated using GFI, CFI, and X^2^ values. A better model fit is indicated when GFI and CFI approach 1, and the X^2^ value approaches 0.

**Figure 12:**
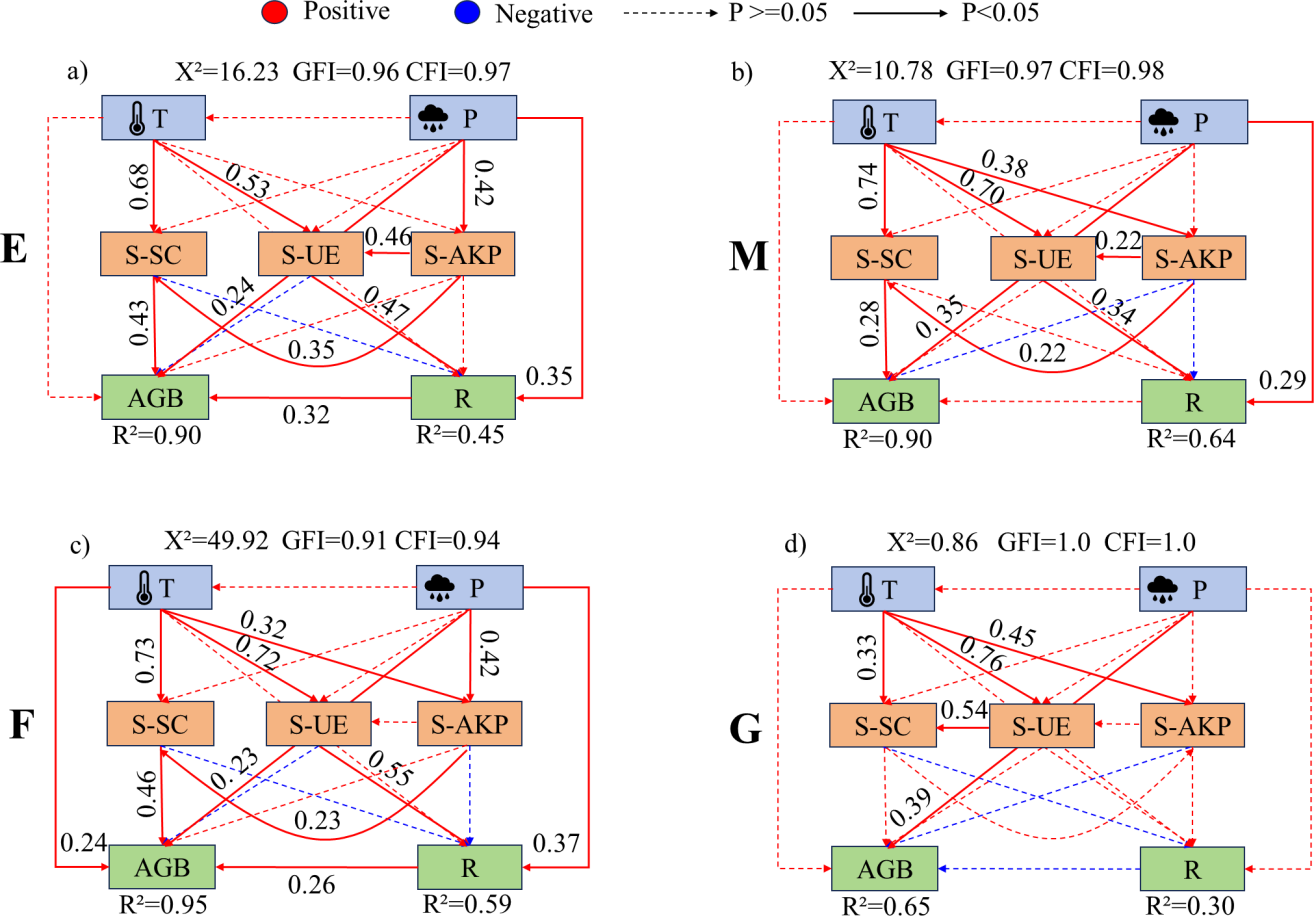
Structural equation model of the effects of climatic factors on soil enzyme activities and vegetation characteristics under different treatments in semi-mobile dunes. **(a-d)** represent the structural equation models illustrating the effects of climatic factors on soil enzyme activity and vegetation under enclosure, mowing, burning, and grazing treatments, respectively. E, Enclosure; M, Mowing; F, Burning; G, Grazing; T, Temperature; P, Rainfall; AGB, Aboveground biomass; R, plant species richness; S-SC, Soil sucrase activity; S-UE, Soil urease activity; S-AKP, Soil alkaline phosphatase activity. Red and blue arrows denote positive and negative relationships, respectively, while solid or dashed lines represent significant (p < 0.05) or non-significant relationships. Path coefficients are indicated near the arrows, showing the standardized path coefficients. R^2^ indicates the proportion of variance explained by each dependent variable. The goodness of fit of the model was evaluated using GFI, CFI, and X^2^ values. A better model fit is indicated when GFI and CFI approach 1, and the X^2^ value approaches 0.

Dune temperature generally had a significant promoting effect on soil enzyme activities (**Figures 10**–**12**). In ecotone dunes, only burning (**Figure 10c**) and grazing treatments (**Figure 10d**) showed a significant positive effect of precipitation on soil sucrase activity (P < 0.001). Temperature significantly promoted aboveground biomass under all treatments in ecotone (**Figure 10**) and fixed dunes (**Figure 11**), whereas in semi-mobile dunes, only burning exhibited a significant promoting effect (P < 0.001; **Figure 12c**). In fixed dunes, precipitation significantly enhanced aboveground biomass only under grazing (P < 0.001; **Figure 11d**). In semi-mobile dunes (**Figure 12**), precipitation significantly promoted aboveground biomass under all treatments (P < 0.001), but its effect on plant species richness was not significant under grazing (P > 0.05; **Figure 12d**).

## 4 Discussion

### 4.1 Responses of vegetation characteristics and soil enzyme activity to different treatments

Our study found that intensive grazing and mowing significantly reduced aboveground biomass and increased plant diversity. Grazing reduces litter input through consumption and trampling, lowering nutrient return and damaging soil structure (Dong et al., 2022). Mowing reduces nutrient compensation by removing biomass, which in turn inhibits plant growth (Hassan et al., 2023). Grazing and mowing promote species coexistence by weakening dominant species and increasing resource availability (Molina et al., 2021). We also found that grazing significantly reduced soil enzyme activity, consistent with the findings of Zhang et al. (2022) in a Stipa grassland. Grazing limits microbial nutrient acquisition by reducing plant productivity, thereby indirectly inhibiting soil enzyme activity (Serrano et al., 2023; Wei et al., 2023).

Mowing shows significant spatial variability in soil enzyme activity. It had no significant effect on soil sucrase and urease activity in the ecotone dune and semi-mobile dune, but significantly reduced soil enzyme activity in the fixed dune. The fixed dune is dominated by Asteraceae, which are less tolerant to mowing. This leads to severe damage to their aboveground parts. After mowing, reduced root exudates from the vegetation inhibit soil enzyme activity (Wang et al., 2012). The vegetation in the ecotone dune is primarily composed of Poaceae, which shows a significant positive response to rainfall (Zhang et al., 2023). The soil in the ecotone dune has higher moisture content, and the suitable soil moisture environment can partially mitigate the negative effects of mowing. Studies have shown that water and nutrient resources are the key factors limiting soil microbial processes (Fan et al., 2026; Liu et al., 2023). Mowing removes aboveground biomass, which leads to a significant decrease in soil moisture (Wang et al., 2023), and reduces soil nutrient deposition (Li et al., 2023). Mowing removes aboveground biomass, leading to a significant decrease in soil moisture and reduced soil nutrient deposition (Li et al., 2023). In the semi-mobile dune, due to extreme scarcity of soil moisture and nutrients, mowing significantly reduced soil alkaline phosphatase activity, which is microbially derived.

Burning has a clear environmental dependence, being jointly regulated by drought intensity and community type (Liu et al., 2022; Orndahl et al., 2022). In the ecotone dune, burning has a minimal effect on vegetation biomass, while in the fixed and semi-mobile dunes, it significantly reduces biomass. This may be due to stronger water stress and weaker vegetation resilience in the latter two dune types. Burning promotes plant species richness in dunes, but this effect was not observed in the semi-mobile dunes. Burning can promote plant species richness by altering interspecies competition (Luo et al., 2024), but in the semi-mobile dunes, long-term natural disturbances hinder community renewal, making it difficult for new species to successfully establish. Burning significantly increased soil enzyme activity in the ecotone dune, significantly decreased it in the semi-mobile dune, and had no significant effect on the fixed dune. Burning affects soil enzyme activity by regulating nutrient cycling and microbial processes. Specifically, burning promotes organic matter mineralization, enhancing nutrient availability and increasing soil enzyme activity (Meng et al., 2020). However, the high temperatures generated during burning simultaneously suppress microbial activity, inhibiting soil enzyme activity (Singh et al., 2021). Therefore, the effect of burning on soil enzyme activity is a result of both damage and compensation. In the semi-mobile dunes, due to water scarcity, fragile soil structure, and strong wind erosion, the organic layer is significantly lost after burning (Fernández-García et al., 2021), leading to a significant decrease in soil enzyme activity. In the ecotone dunes, better moisture conditions help Poaceae roots utilize nutrients released from the burned ashes, offsetting the negative effects of high temperatures on microbes, even leading to a microbial overcompensation effect. The fixed dunes show a balance between damage and compensation, with overall effects reflecting a relative equilibrium.

### 4.2 Relationship between vegetation, soil enzyme activity, and climatic factors under different treatments

#### 4.2.1 Relationship between vegetation characteristics and climatic factors under different treatments

In this study, mowing and burning enhanced the positive correlation between vegetation characteristics and temperature, while grazing had an inhibitory effect. Mowing removes the dominance of plant apices, increases the community’s efficiency in utilizing light resources, and improves surface microclimate conditions. Moderate warming promotes vegetation photosynthesis, thereby enhancing biomass accumulation and the germination of new species (Hassan et al., 2023; Wang et al., 2022a). Burning promotes organic matter mineralization and nutrient release, creating a synergistic effect with temperature, which further enhances vegetation growth and renewal (Xu et al., 2022a, Xu et al., 2022b). Vegetation type is an important factor influencing the relationship between vegetation and rainfall (Chen et al., 2020). In the ecotone and semi-mobile dunes, mowing and burning enhanced the relationship between vegetation and rainfall. This is primarily related to the root distribution characteristics of dominant plants and their compensatory growth ability after disturbance. Poaceae plants have shallow, dispersed fibrous roots that can quickly utilize surface water pulses from rainfall and exhibit strong compensatory growth after disturbance (McAuliffe et al., 2022). In the semi-mobile dunes, shrubs and herbaceous plants coexist. Different life-form species utilize water layers in the soil profile, enhancing the community’s ability to respond to rainfall variation. In contrast, continuous grazing and trampling from grazing intensify vegetation damage, reducing productivity and limiting the ability to regenerate and repair damaged tissues (Hempson et al., 2022). As a result, vegetation prioritizes energy resources for tissue repair, weakening its relationship with climatic factors.

#### 4.2.2 Relationship between soil enzyme activity and climatic factors under different treatments

This study found that mowing and grazing enhance the relationship between enzyme activity and temperature, while weakening its relationship with rainfall. Different disturbances affect the relationship between soil enzyme activity and climatic factors by regulating soil water and thermal conditions. Specifically, mowing and grazing remove aboveground biomass and litter, reducing ground cover, which makes the soil more sensitive to environmental changes, leading to increased soil temperature and decreased soil moisture (Wang et al., 2023; Zhu et al., 2024b). Changes in water and thermal conditions not only affect the root exudation process of vegetation but also regulate the microbial habitat, thereby influencing soil enzyme activity (Chen et al., 2022; Fanin et al., 2022; Shen et al., 2020). In this process, the correlation between soil enzyme activity and temperature strengthens, while water limitation intensifies, weakening the relationship between enzyme activity and rainfall. In contrast, burning affects water and thermal conditions differently by altering surface energy characteristics. On one hand, burning removes vegetation and surface organic matter, reducing albedo and increasing thermal conductivity, allowing the soil to absorb more solar radiation and thus raising surface temperature (Zhao et al., 2021). On the other hand, the ashes produced by burning may block soil pores and increase hydrophobicity, inhibiting water infiltration and leading to increased surface moisture (Li et al., 2022). Under these conditions, soil enzyme activity shows a stronger correlation with both temperature and rainfall.

#### 4.2.3 Relationship between vegetation and soil enzyme activity under different treatments

In this study, grazing weakened the relationship between vegetation and soil enzyme activity. Grazing reduces aboveground biomass and vegetation cover through consumption and trampling, and damages surface soil structure, leading to increased bulk density, decreased infiltration capacity, and intensified wind erosion. This limits microbial access to nutrients, weakening the relationship between vegetation and soil enzyme activity (Zhang et al., 2022). This suggests that vegetation damage, soil structure degradation, and water limitation during grazing are key factors influencing the vegetation-microbe relationship. In the fixed and semi-mobile dunes, burning strengthened the relationship between vegetation and soil enzyme activity. Burning promotes nutrient accumulation and microbial activity in the short term by burning surface biomass, enhancing vegetation recovery in the subsequent growing season (Gao et al., 2023; Hu et al., 2021). Additionally, burning alters surface energy conditions and soil microclimates (Pei et al., 2023), increasing nutrient supply and promoting microbial activity (Qu et al., 2022), thus strengthening the relationship between vegetation and soil enzyme activity. However, in the ecotone dunes, burning weakened the correlation with alkaline phosphatase. This may be due to the soil pH in this area being in the neutral to alkaline range, where inorganic phosphorus readily binds with calcium to form insoluble forms, reducing its availability (Souza-Alonso et al., 2024), thereby limiting the relationship between vegetation and enzyme activity.

This study shows that mowing weakened the relationship between vegetation and soil enzyme activity in the ecotone dunes, but strengthened it in the semi-mobile dunes. Although mowing does not directly damage the roots, it indirectly affects root function by altering the aboveground growth and soil environment (Chen et al., 2021). After mowing, vegetation reallocates auxins to the roots, promoting root growth and recovery (Cui et al., 2021), which changes the root exudation process and microbial carbon supply. In the ecotone dunes, where vegetation growth is vigorous, mowing results in significant aboveground biomass loss. Although Poaceae roots have strong compensatory abilities, the relationship between aboveground vegetation and enzyme activity is weakened. In contrast, in the semi-mobile dunes, possibly due to deeper shrub root distribution and stronger tillering ability, increased root exudates after mowing provide more carbon sources for microbes, enhancing the relationship between vegetation and soil enzyme activity.

### 4.3 Response of soil enzyme activity and vegetation growth to climatic factors under different treatments

This study found that temperature significantly promotes soil enzyme activity, consistent with the findings of Li et al. (2024a) and Zhao et al. (2023). This is primarily because higher temperatures enhance soil respiration, promote root exudation, and accelerate organic matter decomposition, thus speeding up nutrient cycling and enzyme synthesis (Fanin et al., 2022). In contrast, the effect of rainfall on soil enzyme activity shows clear context dependence. In the ecotone dunes, under burning and grazing treatments, rainfall significantly promoted sucrase activity. After burning, the release of ash and active organic carbon increased soil carbon substrate supply (Delijani et al., 2022; Yang et al., 2024). Rainfall improves water-thermal conditions, enhancing the binding efficiency between enzymes and substrates, thereby promoting the carbon cycling process (Song et al., 2024). Meanwhile, in the ecotone dunes, Poaceae plants, which are highly palatable to livestock, suffer from long-term grazing and trampling, leading to increased soil bulk density and reduced porosity. This makes it easier for moisture to accumulate at the surface after rainfall, providing a suitable environment for microbial activity (Guo et al., 2021).

Temperature significantly promotes aboveground biomass in both the ecotone and fixed dunes, which is related to the high stability of vegetation communities, well-developed roots, and favorable soil conditions in these dunes. Warming enhances vegetation photosynthesis and extends the growing season, increasing resource use efficiency and promoting vegetation growth (Yao et al., 2022). In contrast, the semi-mobile dunes are limited by water and nutrients, but burning, by increasing nutrient availability, partially promotes vegetation recovery (Garcia‐Pausas et al., 2022). The effect of rainfall on vegetation is also regulated by environmental conditions and disturbance type. In the fixed dunes under grazing treatment, rainfall promotes vegetation growth, primarily due to increased nutrient input and accelerated litter decomposition (Zhu et al., 2024a). Additionally, Poaceae species in the fixed dunes have low palatability to livestock, reducing physical damage. Livestock, through trampling, accelerates litter decomposition and manure input, increasing nutrient supply (Chuan et al., 2018), thereby enhancing vegetation response to rainfall. In the semi-mobile dunes, vegetation shows greater sensitivity to rainfall, mainly due to significant water limitation in the area (Batbaatar et al., 2021).

## 5 Conclusion

This study, combining correlation analysis and structural equation modeling, explored the relationships between vegetation, soil enzyme activity, and climatic factors under different treatments in desertified dune grasslands. In the dune ecosystem, mowing and burning disturbances showed clear moisture dependence on vegetation and soil enzyme activity. In environments with sufficient moisture, disturbances triggered compensatory effects through resource reallocation, while under water-limited conditions, they mostly acted as suppressive forces. Vegetation type plays an important role in regulating the impact of climatic factors on vegetation. Poaceae plants, with shallow and dispersed fibrous roots, can quickly utilize surface water pulses formed by rainfall and show strong compensatory growth after mowing or burning. Different life-form species regulate the relationship between climatic factors and vegetation through differentiated water use strategies. At the same time, disturbances, through surface cover and soil water-thermal conditions, regulate the relationship between climatic factors and soil enzyme activity. Moisture and vegetation type jointly determine the response of vegetation-soil-microbe processes to climatic factors, highlighting the key role of resource limitation in desertified ecosystems. This study provides new insights into the maintenance mechanisms of dune ecosystems under climate change and human disturbance and offers theoretical references for ecological restoration and management.

## Data Availability

The original contributions presented in the study are included in the article/supplementary material. Further inquiries can be directed to the corresponding authors.
